# Supplementary material for: Pervasiveness of Microprotein Function Amongst Drosophila Small Open Reading Frames (SMORFS)
Source: Cells. 2024 Dec 18;13(24):2090. doi: 10.3390/cells13242090 (PMC11674832; doi:10.3390/cells13242090)
Supplement: Supplementary file 1 [file cells-13-02090-s001.zip › Sup FigS1, FigS2.pptx]

## Slide 1
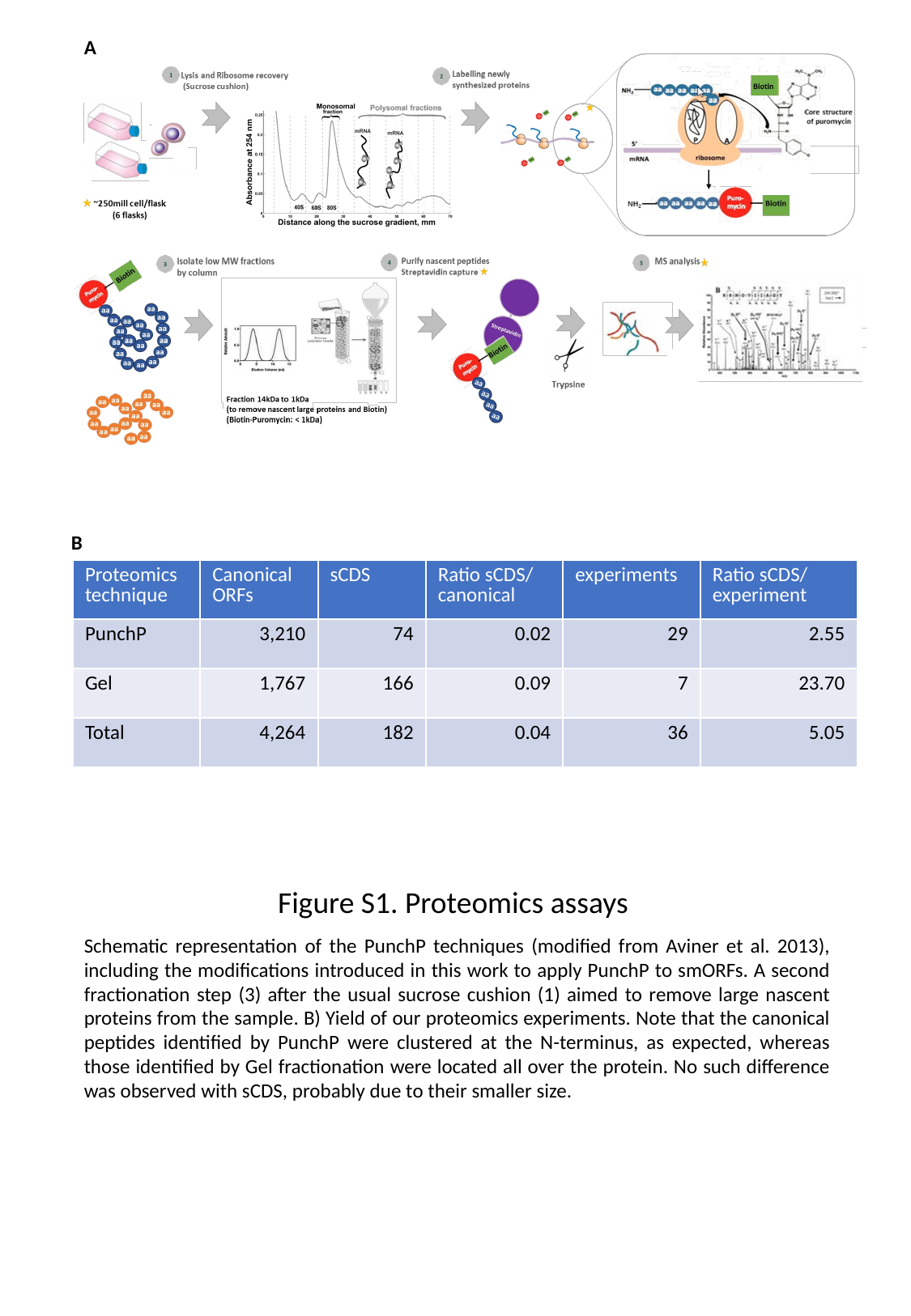

A
B
| Proteomics technique | Canonical ORFs | sCDS | Ratio sCDS/ canonical | experiments | Ratio sCDS/ experiment |
| --- | --- | --- | --- | --- | --- |
| PunchP | 3,210 | 74 | 0.02 | 29 | 2.55 |
| Gel | 1,767 | 166 | 0.09 | 7 | 23.70 |
| Total | 4,264 | 182 | 0.04 | 36 | 5.05 |
Figure S1. Proteomics assays
Schematic representation of the PunchP techniques (modified from Aviner et al. 2013), including the modifications introduced in this work to apply PunchP to smORFs. A second fractionation step (3) after the usual sucrose cushion (1) aimed to remove large nascent proteins from the sample. B) Yield of our proteomics experiments. Note that the canonical peptides identified by PunchP were clustered at the N-terminus, as expected, whereas those identified by Gel fractionation were located all over the protein. No such difference was observed with sCDS, probably due to their smaller size.

## Slide 2
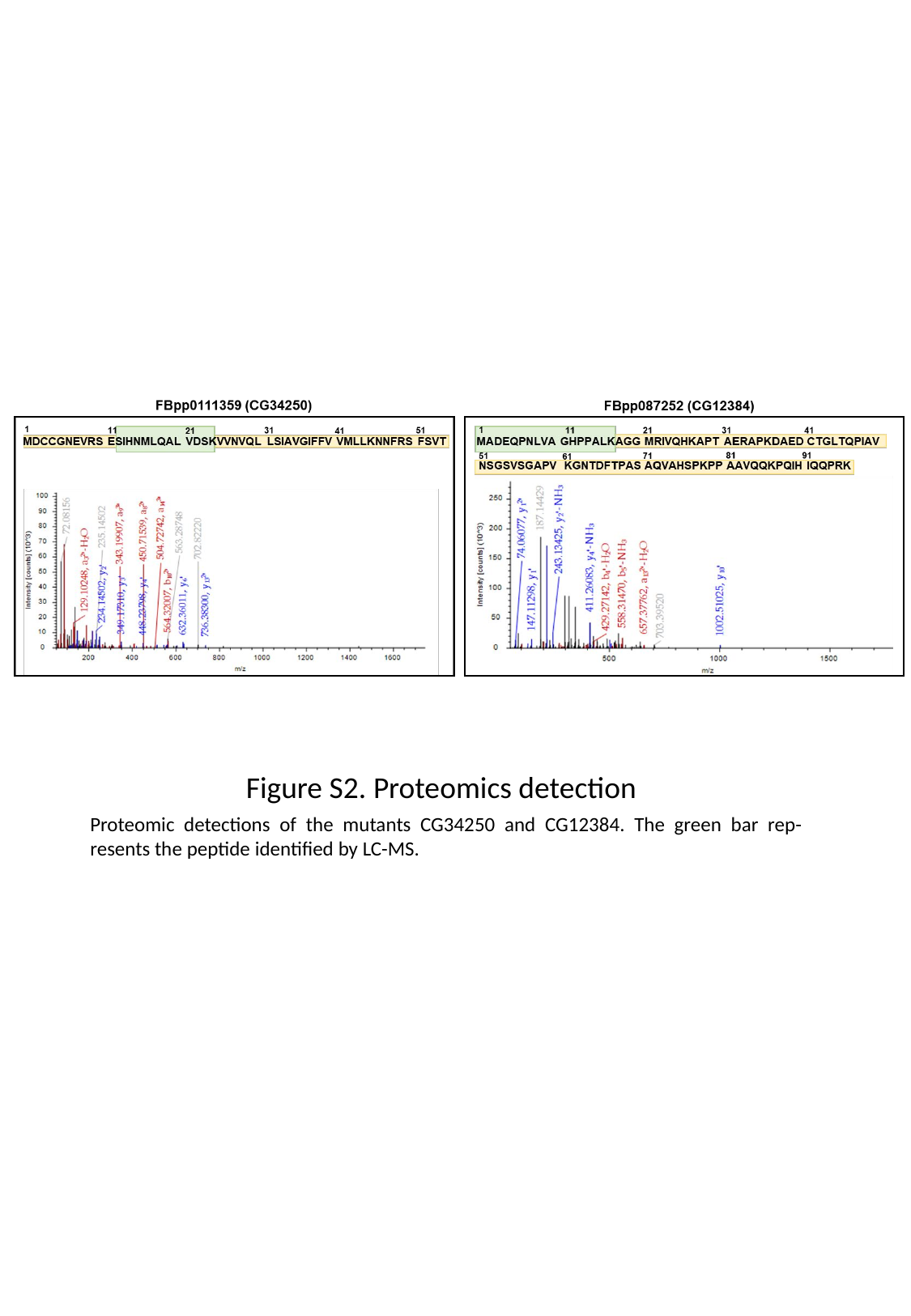

Figure S2. Proteomics detection
Proteomic detections of the mutants CG34250 and CG12384. The green bar rep-resents the peptide identified by LC-MS.
