# Supplementary material for: Pervasiveness of Microprotein Function Amongst Drosophila Small Open Reading Frames (SMORFS)
Source: Cells. 2024 Dec 18;13(24):2090. doi: 10.3390/cells13242090 (PMC11674832; doi:10.3390/cells13242090)
Supplement: Supplementary file 1 [file cells-13-02090-s001.zip › Sup File S3 Embryonic patterns.pptx]

## Slide 1
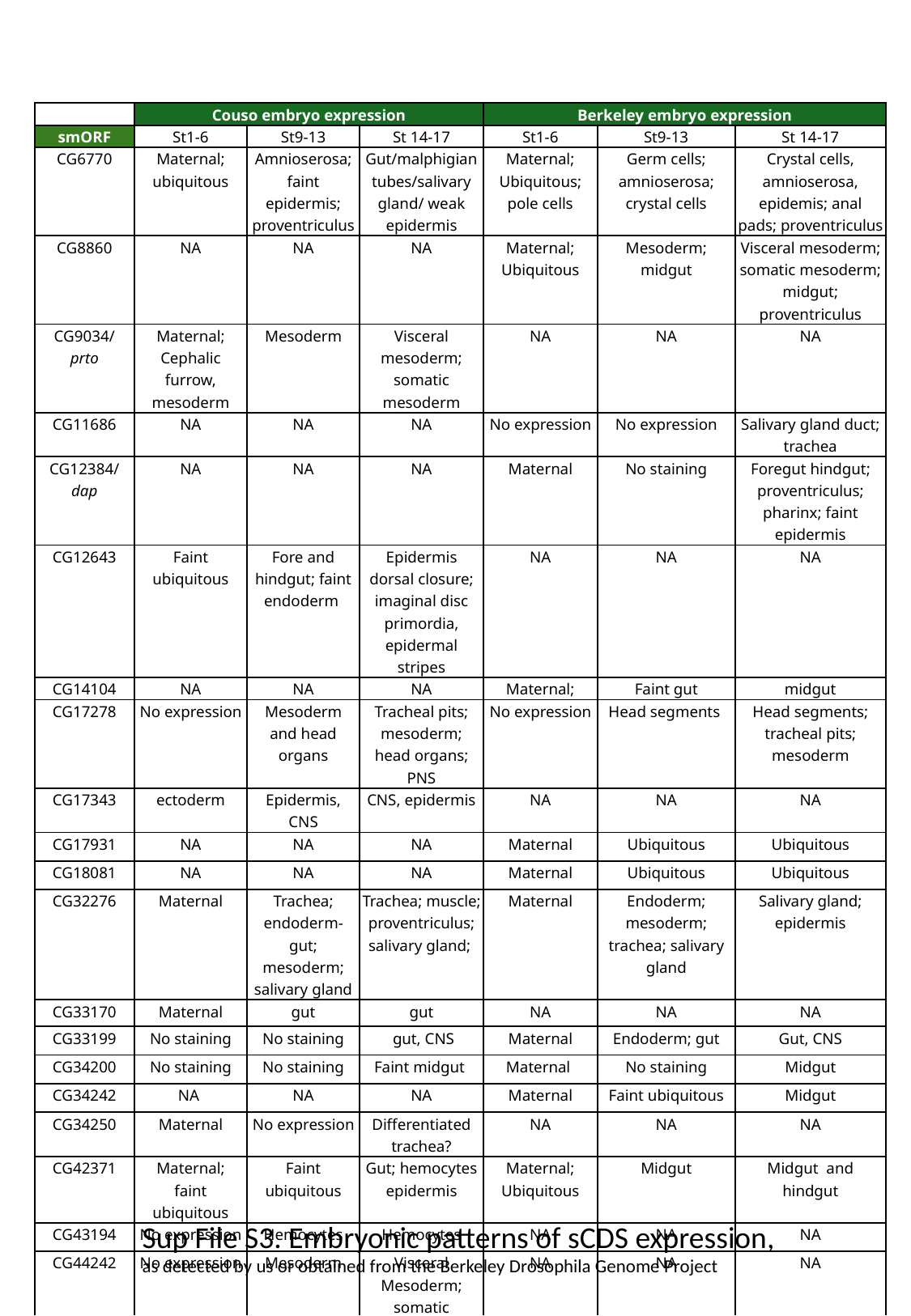

| | Couso embryo expression | | | Berkeley embryo expression | | |
| --- | --- | --- | --- | --- | --- | --- |
| smORF | St1-6 | St9-13 | St 14-17 | St1-6 | St9-13 | St 14-17 |
| CG6770 | Maternal; ubiquitous | Amnioserosa; faint epidermis; proventriculus | Gut/malphigian tubes/salivary gland/ weak epidermis | Maternal; Ubiquitous; pole cells | Germ cells; amnioserosa; crystal cells | Crystal cells, amnioserosa, epidemis; anal pads; proventriculus |
| CG8860 | NA | NA | NA | Maternal; Ubiquitous | Mesoderm; midgut | Visceral mesoderm; somatic mesoderm; midgut; proventriculus |
| CG9034/ prto | Maternal; Cephalic furrow, mesoderm | Mesoderm | Visceral mesoderm; somatic mesoderm | NA | NA | NA |
| CG11686 | NA | NA | NA | No expression | No expression | Salivary gland duct; trachea |
| CG12384/ dap | NA | NA | NA | Maternal | No staining | Foregut hindgut; proventriculus; pharinx; faint epidermis |
| CG12643 | Faint ubiquitous | Fore and hindgut; faint endoderm | Epidermis dorsal closure; imaginal disc primordia, epidermal stripes | NA | NA | NA |
| CG14104 | NA | NA | NA | Maternal; | Faint gut | midgut |
| CG17278 | No expression | Mesoderm and head organs | Tracheal pits; mesoderm; head organs; PNS | No expression | Head segments | Head segments; tracheal pits; mesoderm |
| CG17343 | ectoderm | Epidermis, CNS | CNS, epidermis | NA | NA | NA |
| CG17931 | NA | NA | NA | Maternal | Ubiquitous | Ubiquitous |
| CG18081 | NA | NA | NA | Maternal | Ubiquitous | Ubiquitous |
| CG32276 | Maternal | Trachea; endoderm-gut; mesoderm; salivary gland | Trachea; muscle; proventriculus; salivary gland; | Maternal | Endoderm; mesoderm; trachea; salivary gland | Salivary gland; epidermis |
| CG33170 | Maternal | gut | gut | NA | NA | NA |
| CG33199 | No staining | No staining | gut, CNS | Maternal | Endoderm; gut | Gut, CNS |
| CG34200 | No staining | No staining | Faint midgut | Maternal | No staining | Midgut |
| CG34242 | NA | NA | NA | Maternal | Faint ubiquitous | Midgut |
| CG34250 | Maternal | No expression | Differentiated trachea? | NA | NA | NA |
| CG42371 | Maternal; faint ubiquitous | Faint ubiquitous | Gut; hemocytes epidermis | Maternal; Ubiquitous | Midgut | Midgut and hindgut |
| CG43194 | No expression | Hemocytes | Hemocytes | NA | NA | NA |
| CG44242 | No expression | Mesoderm | Visceral Mesoderm; somatic mesoderm; Malpighian tubes | NA | NA | NA |
Sup File S3. Embryonic patterns of sCDS expression,
as detected by us or obtained from the Berkeley Drosophila Genome Project
